# KI17: A Bioinspired Peptide Derived from *Talisia esculenta* with In Vitro Anticancer and Immunomodulatory Activities

**DOI:** 10.3390/molecules31142434

**Published:** 2026-07-11

**Authors:** Ana Paula Ramos Pereira, Ana Cristina Jacobowski, Camila de Oliveira Gutierrez, Octávio Luiz Franco, Marlon Henrique Cardoso, Thaís de Andrade Farias Rodrigues, Rodrigo Juliano Oliveira, Priscila Aiko Hiane, Rita de Cássia Avellaneda Guimarães, Ana Paula de Araújo Boleti, Maria Lígia Rodrigues Macedo

**Affiliations:** 1 Laboratory of Protein Purification and Biological Functions, FACFAN, Federal University of Mato Grosso do Sul, Campo Grande 79070-900, MS, Brazil; aninha12357@gmail.com (A.P.R.P.); anacristinaj@gmail.com (A.C.J.); camilaogutierrez@gmail.com (C.d.O.G.); apboleti@gmail.com (A.P.d.A.B.); 2 S-Inova Biotech, Graduate Program in Biotechnology, Dom Bosco Catholic University, Campo Grande 79117-900, MS, Brazil; 4899@ucdb.br; 3 Center for Proteomic and Biochemical Analysis, Graduate Program in Genomic Sciences and Biotechnology, Catholic University of Brasília, Brasília 71966-700, DF, Brazil; marlonhenrique6@gmail.com; 4 Graduate Program in Environmental Sciences and Agricultural Sustainability, Dom Bosco Catholic University, Campo Grande 79117-900, MS, Brazil; 5 CeTroGen Laboratory, Universidade Federal de Mato Grosso do Sul, Campo Grande 79070-900, MS, Brazil; tafarias1@gmail.com (T.d.A.F.R.); rodrigo.oliveira@ufms.br (R.J.O.); 6 Faculty of Pharmaceutical Sciences, Food and Nutrition, Universidade Federal de Mato Grosso do Sul, Campo Grande 79070-900, MS, Brazil; priscila.hiane@ufms.br (P.A.H.); rita.guimaraes@ufms.br (R.d.C.A.G.)

**Keywords:** rational peptide design, mitochondrial apoptosis, cell cycle arrest, melanoma

## Abstract

Cancer therapy remains limited by drug resistance and poor selectivity, while inflammation-driven tumor progression further complicates treatment outcomes. Antimicrobial peptides (AMPs) have emerged as promising therapeutic alternatives due to their multifunctional properties. In this study, we investigated the anticancer and immunomodulatory activities of KI17, a rationally designed peptide derived from GL18, a peptide fragment identified from the talisin protein of *Talisia esculenta*. KI17 exhibited dose-dependent antiproliferative effects against murine and human melanoma (B16F10-Nex2, SK-MEL-2, A375) and cervical cancer (HeLa) cell lines, while displaying reduced cytotoxicity toward non-tumoral BV-2 microglial cells, resulting in a favorable selectivity index. Mechanistic analyses revealed that KI17 induces morphological alterations, mitochondrial dysfunction, caspase activation, and late-stage apoptosis, together with G0/G1 cell cycle arrest accompanied by accumulation of the Sub-G0 population, indicating coordinated regulation of cell death and cell cycle progression. KI17 effectively suppressed lipopolysaccharide (LPS)-induced microglial activation, markedly reducing pro-inflammatory cytokine and nitric oxide production without compromising cell viability. These biological activities are consistent with the peptide’s optimized physicochemical features, including increased cationicity, amphipathicity, and α-helical folding. Overall, our findings demonstrate that KI17 combines selective anticancer activity with potent immunomodulatory effects, highlighting its potential as a bioinspired peptide for further preclinical development in cancer therapy.

## 1. Introduction

Cancer remains one of the most significant global public health challenges and a leading cause of premature mortality, representing a major obstacle to increased life expectancy [1]. In 2018, approximately 18 million new cancer cases were diagnosed worldwide, resulting in nearly 10 million deaths. Projections indicate that this number may rise to 29 million new cases by 2040, corresponding to a 63% increase [2,3]. Cancer comprises a heterogeneous group of diseases characterized by uncontrolled proliferation of abnormal cells, arising from complex interactions between genetic and environmental factors, including lifestyle influences [2,3,4].

Carcinogenesis involves the progressive accumulation of genetic and epigenetic alterations, including activation of oncogenes, inactivation of tumor suppressor genes, and dysregulation of pathways controlling genomic stability, apoptosis, and the cell cycle [1,3]. These molecular changes lead to malignant phenotypes such as sustained proliferation, resistance to cell death, angiogenesis, metabolic reprogramming, and metastatic potential, with metastasis representing the main cause of cancer-related mortality [2,5,6].

Although surgery and radiotherapy are effective for localized tumors, chemotherapy remains the standard treatment for advanced disease [7]. However, its clinical use is limited by systemic toxicity, poor selectivity, low bioavailability, and the emergence of multidrug resistance (MDR) [8,9]. Furthermore, tumor cells can develop adaptive resistance mechanisms, including enhanced DNA repair, metabolic reprogramming, and activation of drug efflux systems. In parallel, tumor heterogeneity promotes the selection of resistant subpopulations, contributing to treatment failure and recurrence [2,3].

In this context, cancer is increasingly recognized as a systemic disease involving complex interactions between malignant cells, immune responses, and inflammatory signaling networks [4]. Chronic inflammation plays a key role in tumor progression, angiogenesis, and immune evasion, highlighting the importance of therapeutic strategies capable of simultaneously targeting cancer cells and modulating inflammatory processes [4,6]. Anticancer peptides (ACPs), often derived from AMPs, have emerged as promising candidates because they selectively target tumor cells based on differences in membrane composition [8,10,11]. These peptides are typically cationic and amphipathic, allowing preferential interaction with negatively charged tumor cell membranes, which present exposed phosphatidylserine and other anionic components [12]. In addition to membrane disruption, ACPs can induce mitochondrial dysfunction and activate caspase-dependent apoptosis, processes associated with alterations in mitochondrial membrane permeability and programmed cell death pathways [13,14].

Furthermore, these peptides modulate immune and inflammatory responses within the tumor microenvironment by influencing cytokine production, immune cell activation, and inflammatory signaling pathways [15]. This immunomodulatory activity is particularly relevant, as tumor progression is closely linked to chronic inflammation and immune evasion [4]. By regulating pro- and anti-inflammatory molecules, including key cytokines involved in immune regulation, ACPs can attenuate tumor-promoting inflammation while enhancing antitumor immune responses [15,16]. Collectively, these multifunctional properties make ACPs attractive therapeutic agents with the potential to overcome limitations associated with conventional chemotherapy [16].

In this study, we investigated the anticancer and immunomodulatory potential of KI17, a rationally designed peptide derived from the talisin protein. As previously reported by Pereira et al. 2026 [17], KI17 was developed through targeted sequence optimization aimed at enhancing cationicity, amphipathicity, and α-helical folding. Despite the growing interest in anticancer peptides, many anti-tumor drug candidates still exhibit limited selectivity, reduced stability in biological environments, or insufficient characterization of their immunomodulatory effects. In this study, we evaluate the antiproliferative activity, selectivity, and mechanisms of action of KI17 in tumor cell models, focusing on mitochondrial dysfunction, apoptosis induction, cell cycle arrest, and modulation of inflammatory responses. This study contributes to the development of multifunctional peptide-based therapeutics with improved selectivity and translational potential for cancer treatment.

## 2. Results

### 2.1. Cytotoxic Activity of KI17

The cytotoxic potential of KI17 was assessed using the 3-(4,5-dimethylthiazol-2-yl)-2,5-diphenyl tetrazolium bromide (MTT) assay in five different cell lines: B16F10-Nex2 (murine melanoma), SK-MEL-2 (human melanoma), HeLa (human cervical carcinoma), A375 (human melanoma), and BV-2 (murine microglial cells).

**Figure 1:**
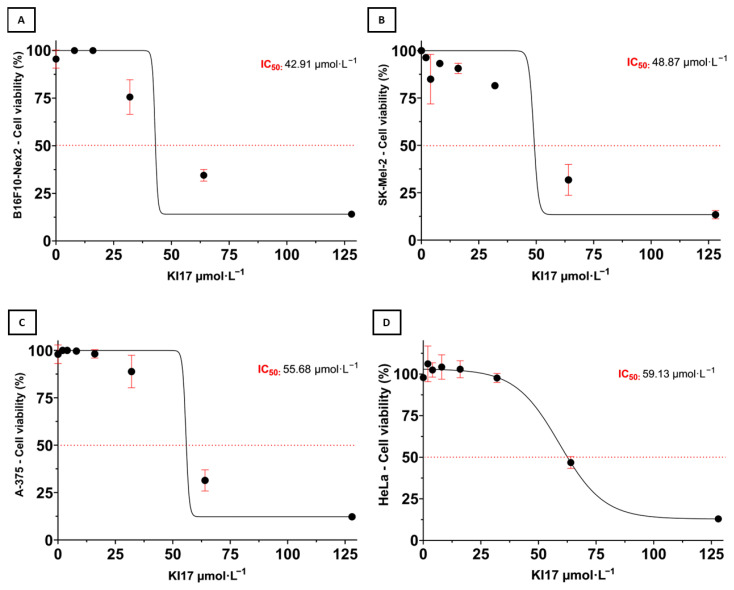
Effect of the KI17 peptide on the viability of tumor cell lines: (**A**) B16F10-Nex2; (**B**) SK-MEL-2; (**C**) A375; and (**D**) HeLa. Cells were treated with increasing concentrations of KI17 for 24 h, and cell viability was assessed using the MTT assay, expressed as a percentage relative to untreated control cells. Dose–response curves were generated by nonlinear regression using a variable-slope model, and the half-maximal inhibitory concentration (IC_50_) was calculated using GraphPad Prism software. The dotted horizontal line indicates 50% cell viability, and the IC_50_ values shown in each panel represent the concentration of KI17 required to reduce cell viability by 50%. Data are presented as mean ± SD from three independent experiments performed in triplicate.

**Figure 2:**
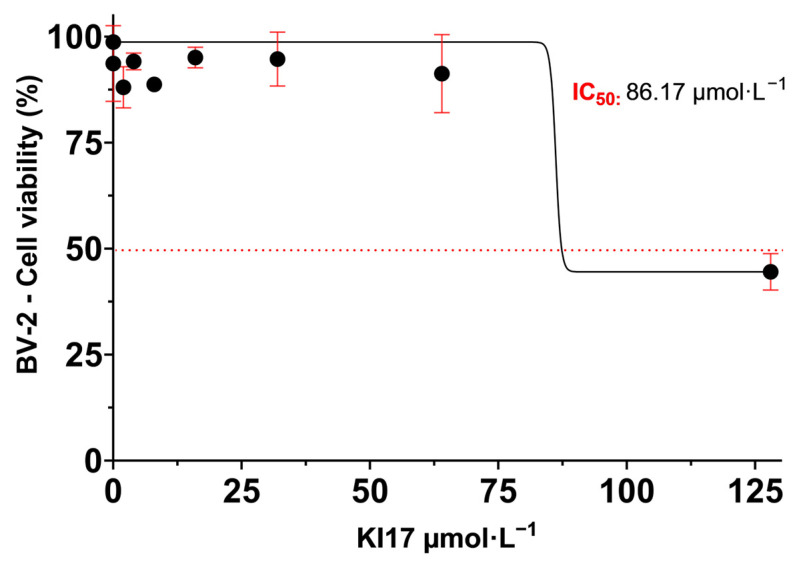
Effect of the KI17 peptide on the viability of the non-tumor cell line BV-2. Murine BV-2 microglial cells were treated with increasing concentrations of KI17 for 24 h, and cell viability was assessed using the MTT assay, expressed as a percentage relative to untreated control cells. The dose–response curve was generated by nonlinear regression using a variable-slope model, and the half-maximal inhibitory concentration (IC_50_) was calculated using GraphPad Prism software. Black dots represent the mean cell viability at each KI17 concentration, and error bars represent the standard deviation (SD). The dotted horizontal line indicates 50% cell viability. Data are presented as mean ± SD from three independent experiments performed in triplicate.

The peptide displayed varying degrees of cytotoxicity across the cell lines, with calculated IC_50_ values of 42.91 µM (B16F10-Nex2, Figure 1A), 48.87 µM (SK-MEL-2, Figure 1B), 55.68 µM (A375, Figure 1C), 59.13 µM (HeLa, Figure 1D), and 86.17 µM (BV-2, Figure 2).

The selectivity index (SI) was calculated as the ratio between the IC_50_ of the non-tumor cell line (BV-2) and the IC_50_ values of the tumor cell lines. KI17 exhibited SI values of 2.01 for B16F10-Nex2, 1.76 for SK-MEL-2, 1.55 for A375, and 1.46 for HeLa, indicating a moderate preferential cytotoxicity toward tumor cells compared with non-tumor BV-2 cells. Given the biological and clinical relevance of the B16F10-Nex2 melanoma model and its pronounced sensitivity to KI17, this cell line was selected for subsequent mechanistic studies to elucidate the peptide’s mode of action.

### 2.2. Cellular Morphological Changes Induced by KI17

**Figure 3:**
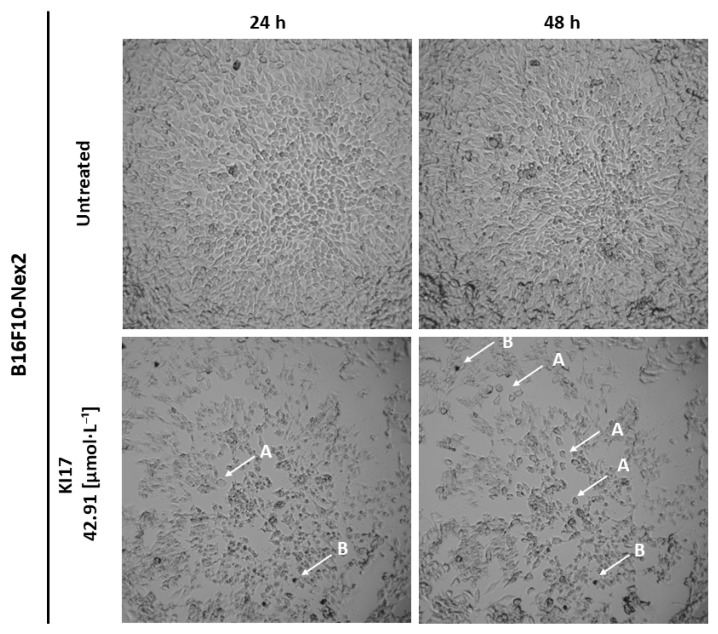
Morphological Changes in B16F10-Nex2 Cells Treated with KI17 Peptide. Phase-contrast images of untreated B16F10-Nex2 cells (control) and cells treated with KI17 peptide (42.91 µmol·L^−1^) for 24 and 48 h. Arrows indicate (A) cell rounding and (B) chromatin condensation, predominantly observed after peptide treatment.

To evaluate the effects of the KI17 on cell morphology, B16F10-Nex2 melanoma cells were monitored for up to 48 h. Phase-contrast images acquired after 24 and 48 h of treatment with KI17 at the IC_50_ concentration revealed progressive morphological changes, compared to control cells (Figure 3).

After 24 h of exposure, cell confluence was reduced, accompanied by morphological alterations, which were characterized by cell rounding and partial loss of cell adhesion to the substrate. After 48 h of treatment, these alterations became more pronounced, with marked disruption of the cell monolayer, an increase in poorly adherent cells, and nuclear alterations that were suggestive of chromatin condensation.

### 2.3. Mitochondrial and Nuclear Changes

**Figure 4:**
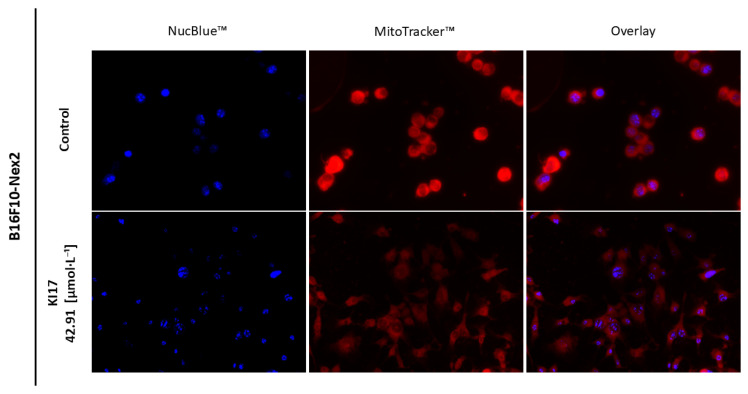
Nuclear and Mitochondrial Morphological Alterations in B16F10-Nex2 Cells after KI17 Treatment at the IC_50_ Concentration (42.91 µmol·L^−1^). Overlay images of B16F10-Nex2 melanoma cells with mitochondria stained in red (MitoTracker™ Deep Red) and nuclei stained in blue (NucBlue™). Images show nuclear swelling and reduced mitochondrial membrane potential following KI17 treatment.

To evaluate the effects of KI17 on mitochondrial membrane potential and nuclear morphology, B16F10-Nex2 melanoma cells were analyzed after 48 h of treatment at the IC_50_ concentration using the MitoTracker™ and NucBlue™ fluorescent probes (Figure 4). Control cells displayed the typical spread morphology, with intensely stained mitochondria homogeneously that were distributed in the cytoplasm, indicating preserved mitochondrial membrane potential and metabolic activity.

In contrast, KI17-treated cells exhibited marked alterations, including a pronounced reduction in mitochondrial fluorescence, suggesting mitochondrial membrane depolarization, while nuclei exhibiting swelling, condensation, and fragmentation. Treated cells also presented cell shrinkage and cytoskeletal retraction, compatible with apoptosis-like morphological features. Based on the time-course analysis across the evaluated time points, 48 h was selected as the most suitable time point for detailed morphological assessment.

### 2.4. Caspase Activation Induced by KI17

**Figure 5:**
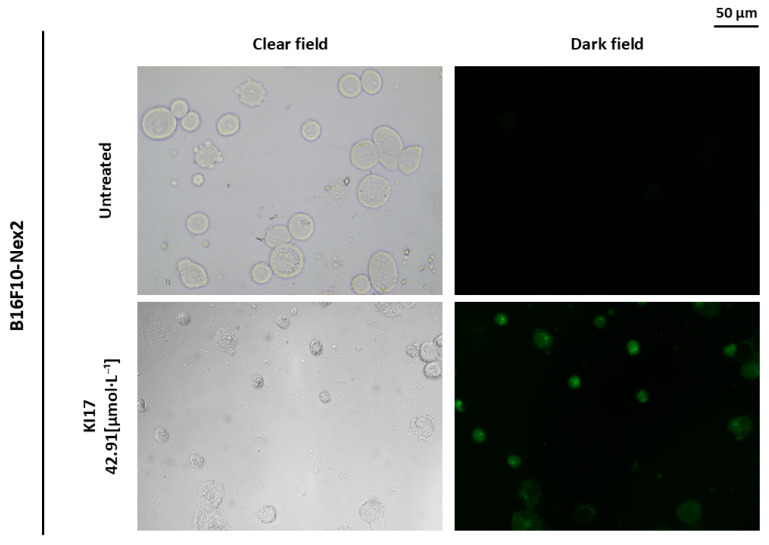
Detection of Active Caspases in B16F10-Nex2 Cells Treated with KI17 Peptide (42.91 µmol·L^−1^). Fluorescence microscopy images of B16F10-Nex2 cells after treatment with KI17 peptide. Green fluorescence indicates activation of caspase pathways in treated cells.

To evaluate whether KI17-induced cell death involves caspase activation, additional assays were performed. Caspase activation was first evaluated in B16F10-Nex2 cells using the fluorescent probe, FITC-VAD-FMK, which binds to active caspases. Fluorescence microscopy revealed a strong green fluorescence signal in KI17-treated cells (Figure 5), indicating caspase activation and suggesting the involvement of apoptosis in the observed cytotoxic effects. In untreated control cells, no detectable fluorescence signal was observed, supporting the conclusion that the fluorescence detected in treated cells resulted from KI17 treatment.

### 2.5. Cell Death Profile Evaluation

**Figure 6:**
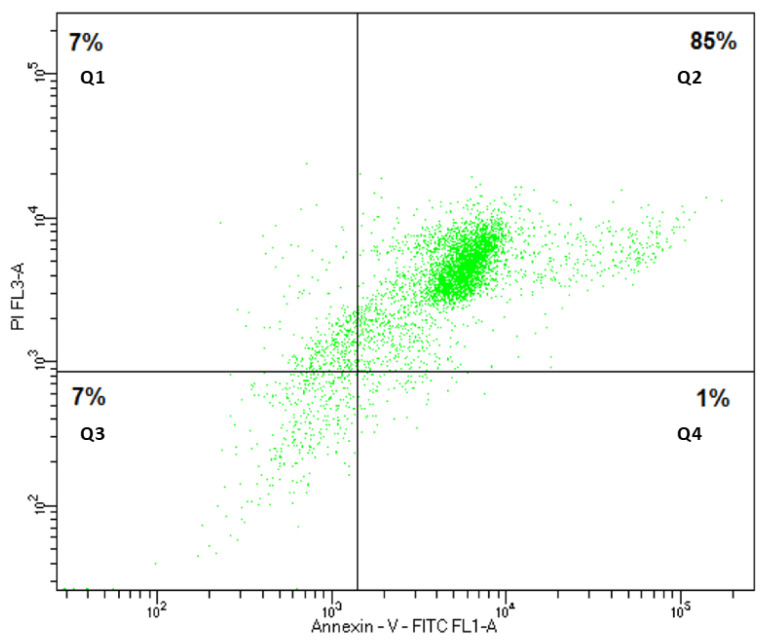
Flow Cytometry Analysis of B16F10-Nex2 Cells Treated with KI17 Peptide (42.91 µmol·L^−1^). Distribution of cell populations across quadrants after Annexin V and propidium iodide staining. Q1, necrotic cells (PI^+^/Annexin V^−^); Q2, late apoptotic cells (PI^+^/Annexin V^+^); Q3, viable cells (PI^−^/Annexin V^−^); Q4, early apoptotic cells (PI^−^/Annexin V^+^). Most cells were located in Q2, indicating late apoptosis.

To further assess the mechanism of cell death induced by KI17, cells were analyzed by flow cytometry using Annexin V-FITC and propidium iodide staining. Most treated cells were distributed in the Q2 quadrant (Annexin V^+^/PI^+^), indicating that approximately 85% of the cells were in late apoptosis (Figure 6). Early apoptotic cells were identified as Annexin V^+^/PI^−^. These results suggest that KI17 predominantly induces late apoptosis in B16F10-Nex2 cells.

### 2.6. Effects of KI17 on Cell Cycle Distribution

**Figure 7:**
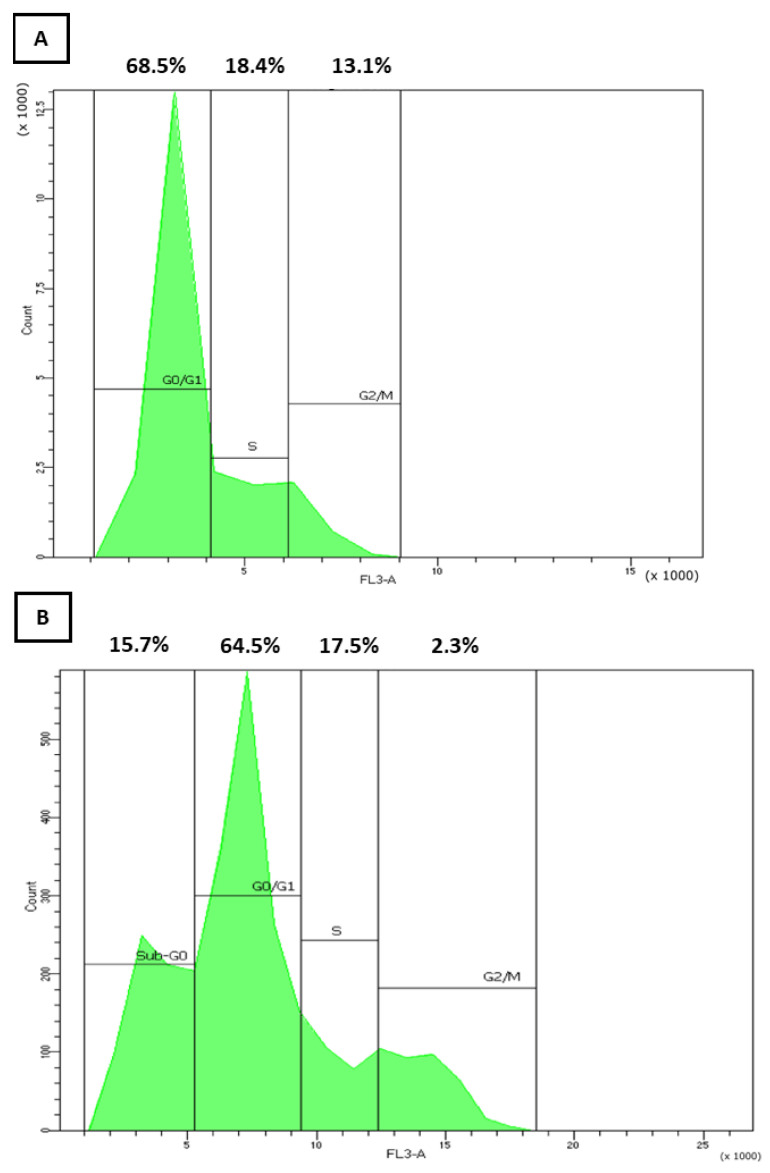
Cell Cycle Distribution of B16F10-Nex2 Cells Treated with KI17. DNA content histograms obtained by flow cytometry after propidium iodide staining, showing the distribution of cells in the Sub-G0, G0/G1, S, and G2/M phases after treatment with KI17 at the IC_50_ concentration. (**A**) Negative control; (**B**) KI17-treated cells.

The cell cycle was analyzed by flow cytometry in control and KI17-treated groups. In the control group, most cells were in the G0/G1 phase (68.5%), followed by the S phase (18.4%) and the G2/M (13.1%) phase, with no significant sub-G0 population detected (Figure 7A).

In the KI17-treated group, the proportion of cells in G0/G1 decreased to 52.2%, compared with the control. The S phase fraction also decreased (11.9%), whereas the G2/M phase remained similar to the control group (14.3%). In addition, a sub-G0 population (21.6%) was detected in the KI17-treated group, which was absent in the control cells (Figure 7B).

### 2.7. Analysis of Microglial Activation and NO Production

**Figure 8:**
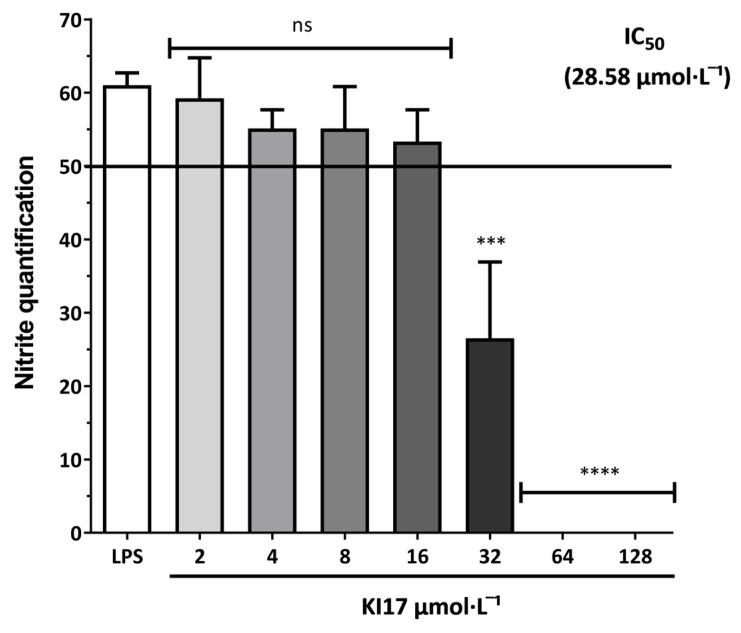
Cellular Nitrite Quantification after Treatment with KI17 Peptide at Different Concentrations. Nitrite levels were measured after treatment with increasing concentrations of KI17, and the IC_50_ value was determined from seven concentrations using nonlinear regression of the dose–response curve. *** *p* < 0.001; **** *p* < 0.0001, compared to untreated cells (ANOVA).

At a concentration of 64 μmol·L^−1^ (Figure 8), KI17 significantly reduced the inflammatory response in LPS-stimulated BV-2 microglial cells. At this concentration, the peptide did not induce significant cytotoxicity, as demonstrated by the MTT assay, indicating that the observed anti-inflammatory effect was not associated with loss of cell viability.

### 2.8. Modulation of Cytokine Production by KI17

**Figure 9:**
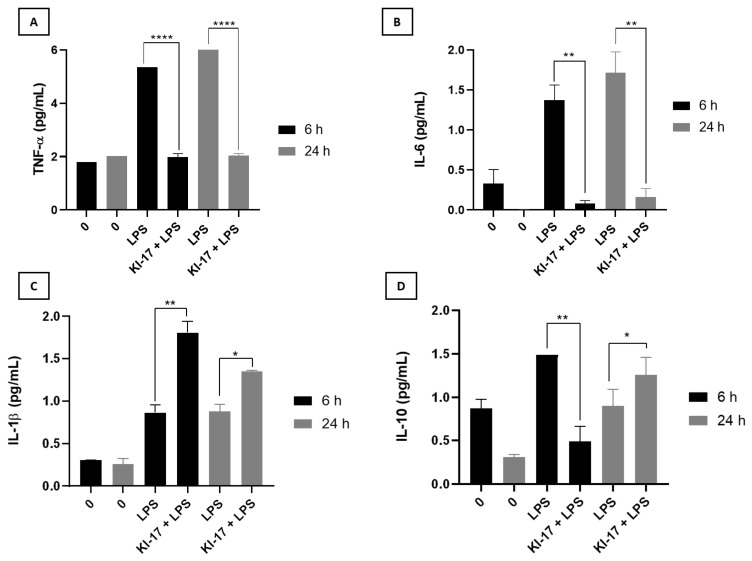
Quantification of Pro- and Anti-Inflammatory Cytokines in LPS-Stimulated BV-2 Cells Treated with KI17 for 6 and 24 h. (**A**) Significant reduction in TNF-α levels; (**B**) marked decrease in IL-6; (**C**) moderate increase in IL-1β; (**D**) elevation of IL-10 levels. Note: The group labeled “0” corresponds to the untreated control, consisting of cells not stimulated with LPS and not exposed to the peptide. Bars represent means ± standard deviation of three independent experiments. Statistical significance was determined in comparison with the untreated control (* *p* < 0.05; ** *p* < 0.01; **** *p* < 0.0001, ANOVA).

KI17 modulated cytokine production in LPS-stimulated BV-2 microglial cells (Figure 9A–D). After 6 and 24 h of treatment, TNF-α levels were reduced by 63% and 70% (Figure 9A), respectively, while IL-6 levels decreased by 94% and 91% (Figure 9B), respectively. These findings suggest a clear inhibitory effect of the peptide on key pro-inflammatory cytokines. In contrast, IL-1β levels increased by 50.6% and 54% after 6 and 24 h (Figure 9C), indicating differential modulation of inflammatory mediators. Additionally, KI17 induced a 40% increase in IL-10 levels at 24 h (Figure 9D), suggesting a potential immunoregulatory effect. Overall, these findings indicate that KI17 modulates the inflammatory response at concentrations that do not compromise cell viability.

## 3. Discussion

Anticancer peptides (ACPs) have emerged as a promising class of biomolecules. These peptides exploit biophysical differences between tumor and normal cells, particularly the increased negative charge of malignant cell membranes that results from the exposure of phosphatidylserine and anionic glycoconjugates [8,16,17]. Frequently derived from antimicrobial peptides (AMPs), ACPs share structural features such as cationic charge and amphipathicity, which favor selective interactions with cellular membranes and the induction of cell death through mechanisms such as mitochondrial dysfunction and caspase activation [13,14,18].

As such, the peptide KI17, which was previously developed through rational design [17], presents physicochemical properties that are typical of cationic amphipathic peptides that interact with biological membranes. Its ability to adopt an α-helical conformation in membrane-mimetic environments favors interaction with anionic surfaces, supporting the hypothesis that KI17 may act not only as an antimicrobial peptide, but also as anticancer peptide. Importantly, the design strategy involved the substitution of residues with low helical propensity by amino acids that promote α-helical stability and amphipathicity, resulting in an increase in net positive charge and hydrophobic moments. These modifications are expected to enhance electrostatic interactions with negatively charged membranes and improve the peptide’s ability to adopt a membrane-active conformation in lipid environments.

The cytotoxic activity of KI17 exhibited a selective profile towards tumor cells, with IC_50_ values ranging from 42.91 to 59.13 µmol·L^−1^, whereas non-tumor BV-2 cells showed lower susceptibility (IC_50_ = 86.17 µmol·L^−1^). This behavior is consistent with that reported for anticancer peptides (ACPs), whose selectivity is often attributed to their preferential interaction with negatively charged membranes, a common feature of malignant cells [13,19].

This difference in sensitivity suggests that KI17 preferentially recognizes cancer cell membranes, which are characterized by higher negative surface potential and lower levels of cholesterol and neutral phospholipids, factors that increase lipid bilayer fluidity and favor interactions with cationic amphipathic peptides [16,19]. In addition, variations among tumor cell lines may reflect not only differences in membrane composition, but also genetic and metabolic characteristics, including the regulation of pathways associated with apoptosis and cellular stress response.

Among the evaluated cell lines, B16F10-Nex2 showed the greatest sensitivity to KI17 and was selected as the experimental model because it represents a well-established metastatic melanoma system, allowing subsequent mechanistic investigations [6,13,20]. The sensitivity of melanoma cell lines is consistent with the biophysical profile of these cells, which present a higher density of anionic lipids and elevated mitochondrial activity, favoring both membrane recognition by amphipathic peptides and activation of energy-dependent cell death pathways [16,19].

The IC_50_ value of KI17 for B16F10-Nex2, a highly metastatic murine melanoma cell line, is in the two-digit micromolar range, similar to that reported for plant-derived bioactive peptides such as the defensin PsD1, isolated from Pisum sativum, which also exhibits cytotoxic activity in melanoma cells at comparable concentrations [21]. In contrast, the AQQSY peptide, derived from cowpea vicilin, presents IC_50_ values in the range of hundreds of micromolar in human tumor cell lines, such as HCT-116, a colorectal cancer cell line [22]. These data indicate that, although bioactive peptides share similar structural characteristics, their cytotoxic potency may vary widely across different cellular models.

Although the selectivity indices obtained for KI17 ranged from 1.46 to 2.01, indicating only moderate selectivity, the peptide consistently displayed lower toxicity toward BV-2 non-tumor cells than toward all evaluated tumor cell lines. This preferential cytotoxicity is consistent with the proposed mechanism of cationic amphipathic peptides, which preferentially interact with negatively charged membranes and may subsequently induce mitochondrial dysfunction and apoptosis [14,16,19]. Similar selectivity profiles have been reported for several anticancer peptides in early-stage investigations [16,19].

The morphological alterations observed after treatment with KI17 reinforce this cytotoxic profile, indicating a progressive process of cell death. Reduced confluence, cell rounding, and loss of adhesion to the substrate, which were more pronounced after 48 h of exposure, are typical features of regulated cell death and have been widely described for amphipathic peptides that act through time-dependent mechanisms, rather than by inducing immediate cell lysis [10].

At the subcellular level, the significant reduction in mitochondrial fluorescence observed in treated cells indicates impairment of the mitochondrial membrane potential (Δψm), a key event in the activation of the intrinsic apoptotic pathway. The dissipation of Δψm is associated with dysfunction of the respiratory chain, increased production of reactive oxygen species, and release of pro-apoptotic factors such as cytochrome c, which culminate in the activation of the caspase cascade [13,14]. This mechanism is frequently described for anticancer peptides that, after their initial interaction with the plasma membrane, can reach intracellular organelles and trigger apoptotic responses.

Corroborating these findings, the activation of caspases detected in KI17-treated cells confirms the involvement of apoptotic pathways in its mechanism of action. This profile is consistent with that observed for bioactive peptides, such as the defensin PsD1, which induces apoptosis in melanoma cells associated with nuclear fragmentation and characteristic morphological alterations [21], as well as the AQQSY peptide, which promotes apoptosis accompanied by modulation of the Bax/Bcl-2 ratio and activation of caspase-3 [22]. These data indicate that caspase-mediated apoptosis is a recurrent mechanism among cationic amphipathic peptides with antitumor activity.

Cell cycle analysis complements these findings, showing a significant increase in the Sub-G0/G1 population after treatment with KI17, reflecting the presence of cells with reduced DNA content resulting from nuclear fragmentation, a classical marker of apoptotic cell death [5,14]. This increase was accompanied by a reduction in the G0/G1 and S phase populations, suggesting that the predominant effect of the peptide is associated with the induction of cell death, rather than specific cell cycle arrest.

This behavior differs from that observed for some bioactive peptides described in the literature, such as AQQSY, which induces cell cycle arrest in the G0/G1 phase [22]. These differences indicate that, although they share similar structural characteristics, cationic amphipathic peptides may act at different stages of cellular regulation, either by directly inducing cell death or by interfering with cell cycle progression.

The effects of KI17 on LPS-stimulated BV-2 microglial cells indicate that, in addition to selective cytotoxic activity in tumor cells, the peptide also plays an important role in modulating the inflammatory response. The significant reduction in nitrite production, without affecting cell viability, suggests that KI17 is capable of attenuating microglial activation without inducing cytotoxicity, an essential aspect for potential therapeutic applications in inflammatory and tumor contexts [5,14].

The modulation of cytokine production, including a marked reduction in pro-inflammatory mediators such as TNF-α and IL-6 throughout the experimental period, reinforces this immunomodulatory profile. These cytokines play a central role in amplifying the inflammatory response and are frequently associated with tumor progression, angiogenesis, and immune evasion [15,19]. The ability of KI17 to suppress these mediators demonstrates an anti-inflammatory effect under LPS-stimulated conditions. Since TNF-α and IL-6 are key mediators involved in inflammatory signaling and have been associated with tumor progression and immune regulation [4,13,15], these findings support the potential immunomodulatory activity of KI17. However, the relevance of these effects within the tumor microenvironment remains to be further investigated in more specific cancer-associated inflammation models.

On the other hand, the increase observed in IL-1β levels indicates that the peptide may also modulate components of the innate immune response, possibly stimulating early inflammatory activation pathways. IL-1β is a key cytokine in the initial inflammatory response, promoting the activation and recruitment of immune cells [13]. This effect, when considered together with the reduction of TNF-α and IL-6, suggests a differential modulation of the immune response rather than global suppression, a feature commonly associated with bioactive peptides that have immunoregulatory properties [13,15].

Additionally, the increase in IL-10 observed after 24 h reinforces the potential of KI17 to promote anti-inflammatory mechanisms and the resolution of inflammation. IL-10 plays a central role in limiting excessive inflammatory responses by inhibiting the production of pro-inflammatory cytokines and regulating immune cell activity [15]. This profile suggests that KI17 may contribute to the balance between pro- and anti-inflammatory responses, favoring a more controlled inflammatory environment [13,15].

It is important to note that the anti-inflammatory effects observed in this study were evaluated using an LPS-stimulated BV-2 microglial model, which represents an exogenous inflammatory stimulus and does not fully reproduce the complexity of endogenous inflammatory processes associated with the tumor microenvironment [4,13]. In addition, cationic amphipathic peptides are known to interact with bacterial lipopolysaccharides and cellular membranes through electrostatic interactions [8,11]. Therefore, part of the reduction in cytokine production observed here may result from partial neutralization of LPS, reducing its effective stimulation of BV-2 cells. Thus, although KI17 clearly modulated inflammatory mediators under the experimental conditions employed, further studies using tumor-associated inflammatory models will be required to determine whether these effects directly reflect modulation of cancer-related inflammatory pathways [4,13,15].

These results suggest that the biological activities observed for KI17 may be related, at least in part, to the rational design strategy employed during its development. The increase in net positive charge and amphipathicity likely favored membrane interactions, which may help explain its selective cytotoxicity, mitochondrial effects, and immunomodulatory profile. Collectively, the findings indicate that KI17 possesses a multifunctional biological profile and support further investigations to clarify its mechanisms of action and potential therapeutic applicability in cancer-related settings.

## 4. Material and Methods

### 4.1. Peptide Synthesis and Preparation

The KI17 peptide was obtained as previously described by Pereira et al., 2026 [17]. The peptide was synthesized by solid-phase peptide synthesis, with ≥95% purity and C-terminal amidation, and was dissolved in sterile water for the cellular assays. Detailed information regarding peptide design, in silico predictions, and physicochemical characterization are described in the original publication.

### 4.2. Anticancer Activity and Cellular Mechanisms (In Vitro)

#### 4.2.1. Cell Culture

Murine melanoma (B16F10-Nex2), human melanoma (SK-MEL-2 and A-375), cervical cancer (HeLa), and murine microglia (BV-2) cell lines were obtained from the Cell Bank of Rio de Janeiro (BCRJ, Rio de Janeiro, Brazil). All cell lines were cryopreserved in liquid nitrogen at approximately −196 °C at the Laboratory of Protein Purification and Their Biological Functions, Federal University of Mato Grosso do Sul (UFMS, Campo Grande, MS, Brazil).

B16F10-Nex2 and BV-2 cells were maintained in RPMI-1640 medium, while SK-MEL-2, A-375, and HeLa cells were cultured in DMEM. Both media were supplemented with 10% fetal bovine serum, 100 U·mL^−1^ penicillin, and 100 µg·mL^−1^ streptomycin (Gibco, Grand Island, NY, USA). All cell lines were maintained at 37 °C in a humidified atmosphere containing 5% CO_2_.

#### 4.2.2. Cell Viability Assays

Cell viability was assessed based on metabolic activity using the MTT colorimetric assay (3-(4,5-dimethylthiazol-2-yl)-2,5-diphenyl-2H-tetrazolium bromide), as described by Mosmann, 1983 [23]. B16F10-Nex2, SK-MEL-2, A-375, HeLa, and BV-2 cells were seeded in 96-well plates at densities of 1 × 10^4^ cells·mL^−1^ for tumor cell lines and 1 × 10^6^ cells·mL^−1^ for BV-2 cells. After 24 h of cell adhesion, cells were treated with KI17 peptide at concentrations ranging from 1 to 128 µmol·L^−1^, diluted in sterile water, and incubated for 24 h at 37 °C in a 5% CO_2_ atmosphere. Subsequently, the medium was removed and 100 µL of MTT solution (1 mg·mL^−1^) were added to each well, followed by incubation for 4 h. The MTT solution was then discarded, and 100 µL of dimethyl sulfoxide were added to dissolve the formazan crystals. Absorbance was measured at 570 nm using a Varioskan Lux microplate reader (Thermo Fisher Scientific, Vantaa, Finland), and cell viability was calculated using SkanIt 6.0 software.

Three independent experiments were performed in triplicate. Cell viability was calculated using the following formula: Cell viability (%) = (Absorbance of sample/Absorbance of negative control) × 100. The half-maximal inhibitory concentration (IC_50_) was determined by nonlinear regression analysis using a four-parameter logistic (4PL) dose–response model (variable slope) implemented in GraphPad Prism 8.0 software (GraphPad Software, San Diego, CA, USA), according to the following equation:

$$Y=Bottom+\frac{Top-Bottom}{1+10^{(LogIC_{50}-X)\times HillSlope}}$$

where *Y* is the observed cell viability (%), *Bottom* and *Top* represent the lower and upper asymptotes of the fitted curve, *X* is the base-10 logarithm of the KI17 concentration, *LogIC*_50_ is the base-10 logarithm of the concentration producing 50% inhibition, and *HillSlope* is the Hill slope (slope factor) of the fitted curve. The selectivity index was calculated as the ratio between the IC_50_ for BV-2 cells and that of each tumor cell line.

### 4.3. Analysis of Cellular Morphological Changes

The morphological analysis assay allows the evaluation of changes that occur during treatment, including alterations related to membrane integrity and early signs of potential cell death. For this assay, B16F10-Nex2 cells were seeded in 24-well microplates (5 × 10^4^ cell·well^−1^) and, when cells reached approximately 90% confluence, treated with KI17 at the IC_50_ concentration (43.57 µM). The plate was incubated at 37 °C in a CO_2_ incubator for 48 h. Cells were monitored by capturing images every 5 min using the ZenCELL Owl 24-channel microscope.

### 4.4. Analysis of Mitochondrial and Nuclear Changes

The evaluation of nuclear and mitochondrial morphological alterations in B16F10-Nex2 cells was adapted from the protocol of Wodlej et al., 2019 [24]. The cells were seeded in 24-well microplates (5 × 10^4^ cell·well^−1^) containing circular coverslips and allowed to reach approximately 80% confluence. The cells were treated with 500 µL of KI17 at the IC_50_ concentration, diluted in RPMI-1640 medium, and incubated for 30 min, 1, 2, 6, 12, 24, and 48 h.

After incubation, the cells were washed with PBS and fixed with 1% paraformaldehyde for 15 min. To stain the mitochondria, 500 µL of RPMI-1640 medium containing MitoTracker Deep Red (Molecular Probes Inc., Eugene, OR, USA) (excitation wavelength 650 nm; emission wavelength 668 nm) at 50 µM were added to each well and incubated for 10 min. Subsequently, to stain the nucleus, a drop (~5 µL) of NucBlue Live ReadyProbes Reagent (Molecular Probes Inc., Eugene, OR, USA) (excitation wavelength 359 nm; emission wavelength 461 nm) was added to each well (~5 µL per well) and incubated for 5 min. The coverslip was mounted with glycerol and observed using a Leica DM 2000 LED microscope equipped with a Leica DFC 7000 T camera.

### 4.5. Detection of Active Nonspecific Caspases in Cells

The B16F10-Nex2 cells were cultured in 12-well plates (5 × 10^4^ cell·well^−1^) containing RPMI-1640 medium without FBS and incubated for 24 h. After reaching approximately 80% confluence, the medium was replaced with fresh RPMI-1640 containing KI17 peptide at its IC_50_ concentration, followed by incubation for an additional 24 h. Cells cultured only in RPMI-1640 medium were used as the negative control. After the incubation period, the medium was removed, and the cells were washed with PBS. Subsequently, 500 µL of CaspACE^TM^ FITC-VAD-FMK In Situ Marker diluted in RPMI-1640 were added to each well at a final concentration of 10 µM, followed by 20 min of incubation in the dark. The medium was then removed, and the cells were washed twice with PBS. After the final wash, the cells were collected, homogenized, and resuspended in PBS, transferred to microtubes, and centrifuged at 7000 rpm for 1 min. The supernatant was removed, and the pellet was resuspended in 100 µL of PBS. Subsequently, an aliquot was then mounted on a glass slide and covered with a coverslip. Fluorescence analysis was performed using a Leica DM 2000 LED microscope equipped with a Leica DFC 7000 T camera.

### 4.6. Cell Death Profile Analysis

The cell death profile was determined using the method described by Van Engeland et al. 1998 [25] with a few modifications. B16F10-Nex2 cells were plated in 6-well plates (1 × 10^6^ cell·well^−1^) and cultured in RPMI-1640 medium supplemented with 10% FBS for 24 h. After reaching approximately 80% confluency, the medium was replaced with the KI17 peptide at its IC_50_ concentration for an additional 24 h. After treatment, the cells were washed with PBS, detached, and resuspended in buffer solution (0.1 M Hepes/NaOH (pH 7.4), 1.4 M NaCl, 25 mM CaCl_2_). The suspension was labeled with annexin V-fluorescein isothiocyanate (FITC) and propidium iodide (BD Pharmingen™) according to the manufacturer’s instructions. Cells were incubated for 15 min at room temperature in the dark and, subsequently, 50,000 events per sample were acquired using flow cytometer.

### 4.7. Cell Cycle Analysis

B16F10-Nex2 cells were plated in 12-well plates at a density of 2 × 10^5^ cells per well and incubated for 24 h at 37 °C in a humidified atmosphere with 5% CO_2_. Cells were then treated with the KI17 peptide at its corresponding IC_50_ concentration for an additional 24 h. After treatment, cells were detached with trypsin, washed with PBS, and incubated with 5 µL of RNase (2 mg/mL) at 37 °C for 30 min. Cells were subsequently incubated with 100 µL of lysis buffer and 5 µL of propidium iodide (PI), followed by a 30 min incubation on ice in the dark. Finally, samples were equilibrated at room temperature for 15 min, and 20,000 events per sample were acquired on a BD Accuri^®^ C6 flow cytometer and analyzed using BD Accuri^®^ C6 software [26].

### 4.8. Evaluation of Anti-Inflammatory and Immunomodulatory Activities

#### 4.8.1. Microglial Activation and NO Production

BV-2 microglial cells were seeded in 96-well plates at a density of 1 × 10^6^ cells·mL^−1^ and incubated for 24 h at 37 °C in a humidified atmosphere with 5% CO_2_. After adhesion, the medium was removed and cells were stimulated with LPS, 1 µg·mL^−1^ in the presence or absence of KI17 peptide at concentrations ranging from 0 to 128 µM, in 100 µL of RPMI medium supplemented with 1% fetal bovine serum. Control groups consisted of cells maintained in medium alone or treated with LPS. After 24 h of incubation, supernatants were collected for nitrite quantification, and cell viability was assessed using the MTT assay.

Nitric oxide production was indirectly determined by measuring nitrite levels using the Griess reagent, as described by Green et al., 1982 [27]. Briefly, 100 µL of supernatant were mixed with an equal volume of freshly prepared Griess reagent, composed of 0.1% N-(1-naphthyl) ethylenediamine dihydrochloride and 1% sulfanilamide in 5% phosphoric acid. After 10 min at room temperature, absorbance was measured at 540 nm, and nitrite concentrations were calculated using a sodium nitrite standard curve (3.12 to 200 µM).

#### 4.8.2. Cytokine Quantification by ELISA

BV-2 microglial cells were seeded in 96-well plates (1 × 10^6^ cell·mL^−1^) and incubated for 24 h at 37 °C in a humidified atmosphere containing 5% CO_2_. Following adhesion, the medium was removed, and cells were stimulated with LPS (final concentration 1 μg·mL^−1^) in the presence or absence of the KI17 peptide (64 μmol·L^−1^), in a final volume of 100 μL per well of RPMI-1640 medium supplemented with 1% FBS. Control groups included cells treated with medium alone or with LPS alone. After 24 h of incubation, cell supernatants were collected for cytokine analysis. Mouse-specific ELISA development kits (ABTS-based) from PeproTech were used to quantify cytokine levels in the supernatants, according to the manufacturer’s instructions. The following kits were employed: TNF (Cat. #900-K54K), IL-6 (Cat. #900-K50K), IL-1β (Cat. #900-K47), and IL-10 (Cat. #900-K53). Absorbance was measured at 405 nm using a microplate reader, and cytokine concentrations were determined based on standard curves generated with known concentrations provided in each [15].

### 4.9. Access to Genetic Heritage

Access to the genetic heritage of microorganisms and the plant species used was registered in the National System for the Management of Genetic Heritage and Associated Traditional Knowledge (SISGEN) under the number A297FAA.

### 4.10. Statistical Analysis

Statistical analyses were performed using one-way analysis of variance (ANOVA) followed by Dunnett’s or Tukey’s multiple comparison tests using GraphPad Prism version 8.0.0 for Windows, GraphPad Software, San Diego, CA, USA.

## 5. Conclusions

Taken together, the results of this study demonstrate that peptide KI17 exhibits antitumor activity associated with apoptosis induction. These effects are mediated by mitochondrial dysfunction and caspase activation in cells, as well as cell cycle alterations consistent with DNA fragmentation. In addition, KI17 modulated the inflammatory response in activated microglial cells, reducing pro-inflammatory mediators while promoting a more immunoregulatory cytokine profile under LPS-stimulated conditions. These findings highlight the potential of KI17 as a multifunctional peptide combining selective cytotoxic and immunomodulatory properties, and provide a basis for future studies aimed at clarifying its therapeutic applicability and mechanisms of action in cancer-related contexts.

## Data Availability

The data presented in this study are available on request from the corresponding author.
